# MOG encephalomyelitis after vaccination against severe acute respiratory syndrome coronavirus type 2 (SARS-CoV-2): case report and comprehensive review of the literature

**DOI:** 10.1007/s00415-022-11194-9

**Published:** 2022-06-23

**Authors:** S. Jarius, N. Bieber, J. Haas, B. Wildemann

**Affiliations:** 1 Molecular Neuroimmunology Group, Department of Neurology, University of Heidelberg, Heidelberg, Germany; 2 Department of Neurology, University of Heidelberg, Heidelberg, Germany; 3 Otto Meyerhof Center, Im Neuenheimer Feld 350, 69120 Heidelberg, Germany

**Keywords:** Myelin oligodendrocyte glycoprotein antibody-associated encephalomyelitis (MOG-EM), Myelin oligodendrocyte glycoprotein antibody-associated disease (MOGAD), Severe acute respiratory syndrome coronavirus type 2 (SARS-CoV-2), Coronavirus disease 2019 (COVID-2019), Vaccination, Postvaccinal, ChAdOx1-S, ChAdOx1 nCoV-19, AZD1222, BNT162b2, mRNA-1273, Myelitis, Optic neuritis, Encephalitis, Brainstem encephalitis, Longitudinally extensive transverse myelitis (LETM), Neuromyelitis optica spectrum disorders, MOG antibodies (MOG-IgG)

## Abstract

**Background:**

In around 20% of cases, myelin oligodendrocyte glycoprotein (MOG) immunoglobulin (IgG)-associated encephalomyelitis (MOG-EM; also termed MOG antibody-associated disease, MOGAD) first occurs in a postinfectious or postvaccinal setting.

**Objective:**

To report a case of MOG-EM with onset after vaccination with the Pfizer BioNTech COVID-19 mRNA vaccine BNT162b2 (Comirnaty®) and to provide a comprehensive review of the epidemiological, clinical, radiological, electrophysiological and laboratory features as well as treatment outcomes of all published patients with SARS-CoV-2 vaccination-associated new-onset MOG-EM.

**Methods:**

Case report and review of the literature.

**Results:**

In our patient, MOG-IgG-positive (serum 1:1000, mainly IgG1 and IgG2; CSF 1:2; MOG-specific antibody index < 4) unilateral optic neuritis (ON) occurred 10 days after booster vaccination with BNT162b2, which had been preceded by two immunizations with the vector-based Oxford AstraZeneca vaccine ChAdOx1-S/ChAdOx1-nCoV-19 (AZD1222). High-dose steroid treatment with oral tapering resulted in complete recovery. Overall, 20 cases of SARS-CoV2 vaccination-associated MOG-EM were analysed (median age at onset 43.5 years, range 28–68; female to male ratio = 1:1.2). All cases occurred in adults and almost all after immunization with ChAdOx1-S/ChAdOx1 nCoV-19 (median interval 13 days, range 7–32), mostly after the first dose. In 70% of patients, more than one CNS region (spinal cord, brainstem, supratentorial brain, optic nerve) was affected at onset, in contrast to a much lower rate in conventional MOG-EM in adults, in which isolated ON is predominant at onset and ADEM-like phenotypes are rare. The cerebrospinal fluid white cell count (WCC) exceeded 100 cells/μl in 5/14 (36%) patients with available data (median peak WCC 58 cells/μl in those with pleocytosis; range 6–720). Severe disease with tetraparesis, paraplegia, functional blindness, brainstem involvement and/or bladder/bowel dysfunction and a high lesion load was common, and treatment escalation with plasma exchange (*N* = 9) and/or prolonged IVMP therapy was required in 50% of cases. Complete or partial recovery was achieved in the majority of patients, but residual symptoms were significant in some. MOG-IgG remained detectable in 7/7 cases after 3 or 6 months.

**Conclusions:**

MOG-EM with postvaccinal onset was mostly observed after vaccination with ChAdOx1-S/ChAdOx1 nCoV-19. Attack severity was often high at onset. Escalation of immunotherapy was frequently required. MOG-IgG persisted in the long term.

## Introduction

Myelin oligodendrocyte glycoprotein (MOG) immunoglobulin (IgG)-associated encephalomyelitis [5, 14, 23, 43] (MOG-EM; also termed MOG antibody-associated disease, MOGAD) is a relatively newly described entity. Together with neuromyelitis optica spectrum disorders (NMOSD), MOG-EM is the most important differential diagnosis of multiple sclerosis (MS) [18, 29]. The manifestations include optic neuritis, myelitis, brainstem encephalitis, and encephalitis (with and without seizures). In around 20% of cases, MOG-EM first occurs in a postinfectious or postvaccinal setting [20]. Accordingly, acute disseminated encephalomyelitis (ADEM) is a frequent manifestation of MOG-EM, especially in children. In recent months, almost 20 cases of newly emerging MOG-EM have been reported in which the onset of disease was preceded by vaccination against the severe acute respiratory syndrome coronavirus type 2 (SARS-CoV-2) [6–9, 30, 32, 34, 36]. Here we report the first case of MOG-EM in a Caucasian patient after vaccination with the Pfizer-BioNTech COVID-19 mRNA vaccine BNT162b2. In addition, we provide a review of the epidemiological, clinical, radiological, electrophysiological and laboratory features of all patients with SARS-CoV-2 vaccination-associated MOG-EM published so far and of treatment outcomes.

## Methods

### Case report

Data were retrieved retrospectively from the patient’s records. The patient gave written informed consent for publication.

### Literature review

A PubMed search was performed using the following search term: (“MOG antibody disease” OR “MOG antibody-associated disease” OR “MOG antibody-associated disorder” OR “MOGAD” OR (“mog” AND (“encephalomyelitis” OR “IgG” OR “immunoglobulin g” OR “antibody” OR “antibodies” OR “autoantibody” OR “autoantibodies”)) AND (“COVID-19” OR “COVID19” OR “SARS-CoV-2” OR “SARS-CoV2” OR “severe acute respiratory syndrome” OR “coronavirus”)). Articles published by 1 April 2022 in English, German or French were considered. The following parameters were extracted for each reported patient: age at presentation, sex, origin, vaccine used, number of vaccinations before onset of MOG-EM (one, two, three or four doses), time between latest vaccination and onset of neurological symptoms attributable to MOG-EM, clinical features, magnetic resonance imaging (MRI) features, MOG-IgG serum and cerebrospinal fluid (CSF) status and titres, aquaporin-4 (AQP4)-IgG serostatus, CSF white cell count and cytological results, presence of CSF-restricted oligoclonal IgG bands, CSF total protein, glucose and lactate levels, acute and long-term treatments used, follow-up intervals, outcome, excluded differential diagnoses, history of coronavirus disease 2019 (COVID-19), general and family history.

## Results

### Case report

A 67-year-old Caucasian man with no remarkable medical history except for arterial hypertension and benign prostate hyperplasia and a negative family history for autoimmune disorders experienced an acute decline in visual acuity (OS 0.63, OD 1.0) and colour desaturation in the left eye associated with left-sided temporal headache and pain upon eye movement 10 days after vaccination with Comirnaty®. Two vaccinations with Vaxzevria® had taken place 210 and 154 days earlier. MRI showed swelling and contrast enhancement of the anterior part of the left optic nerve compatible with a diagnosis of optic neuritis (ON) but no further brain or spinal cord (SC) lesions. Visual evoked potentials (VEP) demonstrated prolonged absolute and relative P100 latency and marked amplitude reduction in the left eye (by 40 ms and 73%, respectively, compared with the right eye), suggesting a pre-chiasmic left-sided optic nerve lesion. Ophthalmoscopically, no signs of papillitis were seen. Lumbar puncture demonstrated mild lymphomonocytic pleocytosis (6 white cells/µl; including 1 granulocyte/µl) with signs of monocytic transformation and mildly elevated CSF total protein (51 mg/dl). CSF-restricted oligoclonal IgG bands (OCB) were absent (OCB pattern 1). IgG, IgM, IgG and albumin CSF/serum ratios were normal. Serum MOG-IgG was positive at a titre of 1:2,500 in a fixed CBA using HEK293 cells transfected with full-length human MOG as antigenic substrate and a Fc*γ*-specific secondary antibody (University Hospital Heidelberg, Heidelberg, Germany). A matched CSF sample was positive for MOG-IgG at a titre of 1:2 but was negative when tested at a titre that would indicate intrathecal MOG-IgG synthesis (MOG-specific antibody index [AI] < 4). Serum MOG-IgG belonged mainly to the IgG1 subclass (IgG1 > IgG2 >> IgG4; no IgG3); in a matched CSF sample exclusively MOG-IgG1 antibodies were detected. Serum and CSF AQP4-IgG was negative, as were serum antinuclear antibodies (ANA), antineutrophil cytoplasmic antibodies (ANCA), and rheumatoid factor. Vitamin B12 and angiotensin converting enzyme serum levels, blood sedimentation rate, and AIs for *Borrelia burgdorferi* and *Treponema pallidum* were all normal. MOG-IgG seropositivity was confirmed in a second laboratory (Euroimmun, Lübeck, Germany) and a diagnosis of MOG-EM was made. Treatment with high-dose intravenous methylprednisolone (IVMP) for 3 days (1 g/d) followed by oral tapering (starting at 100 mg/d methylprednisolone) over 44 days resulted in complete recovery (except for residual phosphenes in the dark). VEP were normal at retesting 86 days after onset but MOG-IgG was still detectable, although at lower titre (1:320). At last follow-up, 131 days after onset no new symptoms had occurred.

### Literature review

#### Epidemiology

Including the present case, 20 cases of newly emerging MOG-EM after vaccination against SARS-CoV-2 have been published so far [6–9, 30, 32, 34, 36] (Table 1), with the earliest report dating back to February 2021, i.e., not long after the onset of the vaccination campaign. The median age at onset of MOG-EM was 43.5 years (range 28–68), which is higher than that observed in previous adult cohorts that did not include SARS-CoV-2 vaccination-associated cases (e.g. 36 years in the adult subgroup in [20] [*p* = 0.078], 34 in [19] [*p* = 0.015], 37 years in [43], and 37 years in [5]). The female:male ratio was 1:1.2. The patients were from India (*N* = 10), the UK (*N* = 4), Germany (*N* = 3), Japan (*N* = 2), and Italy (*N* = 1).

**Table 1:**
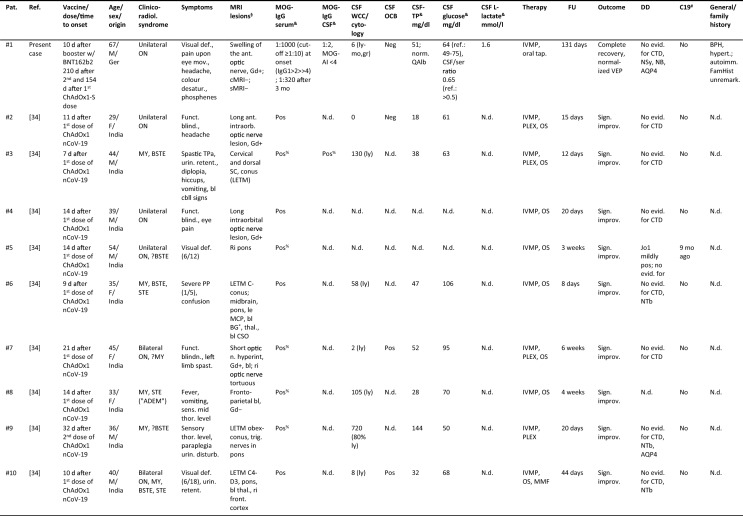

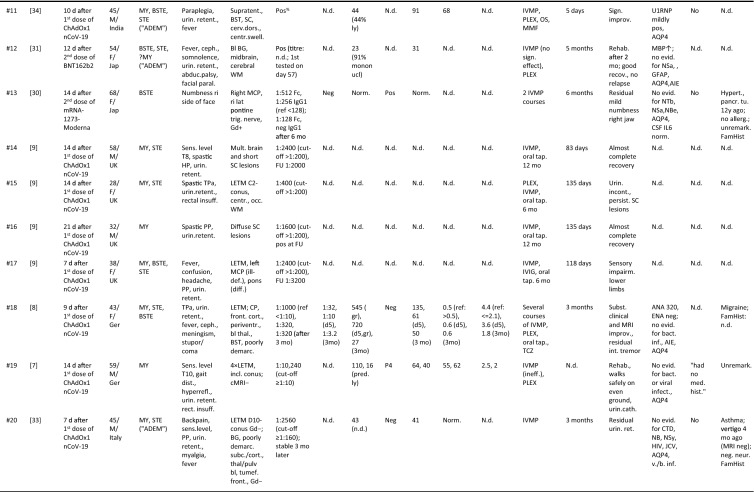
Clinical and paraclinical data in 20 patients with newly emerging MOG-EM after vaccination against SARS-CoV-2

Abbreviations: *ADEM* acute disseminated encephalomyelitis, *AI* antibody index, *AIE* autoimmune encephalitis, *AQP4* AQP4-IgG-positive neuromyelitis optica spectrum disorders, *bl* bilateral, *BG* basal ganglia, *BPH* benign prostate hyperplasia, *BST* brainstem, *BSTE* BST encephalitis, *C* cervical, *C19* coronavirus disease 2019, *cbll* cerebellar, *cMRI* cranial MRI, *CP* cerebellar peduncle, *CSO* centrum semiovale, *CSF* cerebrospinal fluid, *CTD* connective tissue disorders, *D* dorsal (thoracic), *DD* differential diagnostics, *FamHist* family history, *FU* follow-up, *Ger* Germany, *Gd* Gadolinium-enhanced imaging, *GFAP* glial fibrillary astrocytic protein antibody-associated encephalomyelitis, *HIV* human immunodeficiency virus, *HP* hemiparesis, *IL6* interleukin-6, *IVMP* intravenous methylprednisolone, *JCV* John Cunningham virus, *Jap* Japan, *le* left, *LETM* longitudinally extensive transverse myelitis, *MBP* myelin basic protein, *MCP* mid cerebellar peduncle, *MMF* mycophenolate mofetil, *MRI* magnetic resonance imaging, *MY* myelitis, *N.d.* no data, *NB* neuroborreliosis, *NBe* neuro-Behcet, *NSa* neuro-Sarcoidosis, *NTb* neuro-tuberculosis, *OCB* oligoclonal bands, *P4* pattern 4 OCB, *OS* oral steroids, *Pat.* Patient, *PLEX* plasma exchange, *PP* paraparesis, *QAlb* albumin CSF/serum ratio, *ref* reference, *ri* right, *SC* spinal cord, *sMRI* spinal MRI, *STE* supratentorial encephalitis, *TCZ* tocilizumab, *TP* total protein, *TPa* tetraparesis, *UK* United Kingdom, *unil* unilateral, *v./b inf.* viral/bacterial infections, *VEP* visual evoked potential, *WCC* white cell count, *WM* white matter

^§^T2/FLAIR if not indicated otherwise; ^&^first and follow-up measurements in chronological order; ^#^COVID-19 (prior or concomitant); ^%^reported as “strongly pos” (no titre given); ^*^internal capsule

*Associated vaccines.* MOG-EM was directly preceded by vaccination with the vector-based AstraZeneca/Oxford vaccine ChAdOx1-S/ChAdOx1 nCoV-19 (AZD1222) in 17/20 (85%) cases, by vaccination with the mRNA-based Pfizer-BioNTech vaccine BNT162b2 in two cases, and by vaccination with the Moderna vaccine mRNA-1273 in one case. In one of the two BNT162b2-associated cases, vaccination with BNT162b2 followed two previous vaccinations with ChAdOx1-S.

*Time course.* All vaccinations took place within 6 weeks before disease onset (median 13 days, range 7–32, quartile range 10–14). All cases except for the present case, which began after the third (booster) vaccination, occurred after the first (16/20; 80%) or second (3/20; 15%) vaccination.

*Clinicoradiological syndromes.* Clinical symptoms and/or radiological signs compatible with myelitis (MY) were present, alone or in combination, in 15/20 [75%] patients (with no indication of involvement of other CNS areas in only 2 cases), followed by brainstem encephalitis (BSTE) (10/20 [50%]; isolated brainstem [BST] involvement in only one case), and supratentorial encephalitis (STE) (10/20 [50%]; with indication of involvement of other CNS areas in all). The optic nerve was affected in 6/20 (30%) patients (with no indication of involvement of other symptoms in 3/6; bilateral in 2/6) (Table 1). Overall, more than one CNS region (SC, BST, supratentorial brain [STB], optic nerve) was clinically and/or radiologically affected in 14/20 (70%) patients (4 × SC + STB, 4 × SC + BST + STB, 2 × SC + BST, 1 × SC + BST + STB, 1 × SC + BST + STB + optic nerve, 1 × SC + optic nerve, 1 × BST + optic nerve), with at least three regions affected in 6/20 (30%) and all four in 1/20 (5%).

This is in contrast to data from patients with non-postvaccinal MOG-EM. ON was the initial manifestation in 19/26 (73%) adult patients in an Australian cohort [41] and in 34/56 (61%) in a Spanish cohort [43]. In another European cohort, isolated ON was the initial manifestation in 32/48 (67%) of non-vaccination-associated cases (compared with 3/20 or 15% in the present study) [12, 20, 21]. While dissemination in space (here understood as involvement of more than one of the abovementioned anatomical sites) was present at onset in only 8/50 (16%) cases (and only in 16/50 [32%] over the entire observation period) in the latter study, it was present in 70% in the present cohort.

#### Clinical and paraclinical findings: ON

The symptoms in patients with SARS-CoV-2 vaccination-associated MOG-ON were typical for those reported in MOG-ON in general and included blurred vision, decrease in visual acuity, colour desaturation, visual field impairment, pain upon eye movement, headache, and in 6/6 cases a relative afferent pupillary defect. Papillitis, a common finding in MOG-ON, was excluded ophthalmologically in one patient but not reported in the remainder. Of note, however, visual acuity was strongly diminished during acute ON in several patients (1 × hand movement perception close to face; 1 × finger counting at 2 m; 1 × hand movement perception in the left eye and 6/12 m in the right eye, 1 × 6/18 m in both eyes, 1 × 6/12 m in both eyes; 1 × 6/9.5) and VEP were strongly altered. Overall, eight eyes were clinically affected in six patients; VEP revealed an absent P100 wave in 4/6 eyes with available data (no data in one patient with bilateral ON), both prolonged P100 latency (140 ms) and significantly reduced amplitude in one eye, and prolonged P100 latency (132 ms) but no amplitude reduction in one eye (no data available for two eyes). In two further patients with a clinical diagnosis of unilateral ON, P100 latencies were borderline prolonged in the other eye (127 and 115 ms, respectively). MRI demonstrated a longitudinally extensive optic nerve lesion (LEON) in 2/4 patients with available data. The anterior part of the optic nerve was affected in at least three patients, but lesion location was not given in the remainder. Swelling of the optic nerve was explicitly mentioned in one case and optic nerve tortuosity in another one (no data in the remainder). Gadolinium enhancement of the optic nerve was present in 4/4 with available data. Optic coherence tomography data were not reported.

#### Clinical and paraclinical findings: myelitis

Symptoms and signs in those with signs of spinal cord involvement included spastic tetraparesis (3 ×), paraplegia (4 ×), paraparesis (2 ×), left limb spasticity (1 ×), hyperreflexia of the lower limbs without paresis (1 ×), sensory level (4 ×), urinary retention (12 ×), rectal sphincter dysfunction (2 ×), and backpain. Longitudinal extensive transverse myelitis (LETM) was present on MRI at least in 9/10 patients with SC involvement and available data (no data in four). Multiple SC lesions, including multiple LETM lesions, were found in at least six patients. The conus was involved in at least six patients. LETM lesions were particularly long in several patients (3 × obex/cervical SC to conus, 1 × dorsal SC to conus, 1 × C3-D3) and multiple LETM lesions were present in at least two (1 × 4 LETM lesions, 1 × 2 LETM lesions) (no data in two). Gadolinium enhancement was explicitly reported only in two patients and was negative in both (no data in the remainder).

In two patients, short-term follow-up MRI showed an increase in SC lesion load (four LETM lesions instead of one in one case after 5 days [6], and a new LETM and several new brain and BST lesions in another after 5 days [7]), suggesting that early MRI examinations may underestimate the peak lesion load in some cases.

#### Clinical and paraclinical findings: BSTE

Symptoms attributable to BST involvement included bilateral abducens nerve palsy, “diplopia” (not further specified), facial paralysis, hiccups and vomiting (suggestive of possible area postrema syndrome), and cerebellar signs and symptoms. Lesions on MRI involved the pons in 6/10 patients (60%), the mid cerebellar peduncle (CP), a structure often involved in MOG-EM, in 4/10 (40%), the midbrain in 2/10 (20%), and other, non-specified BST areas in another two patients. Gadolinium enhancement was seen in one patient and was not specified in the remainder.

#### Clinical and paraclinical findings: STE

In patients with involvement of the supratentorial brain, fever (at least in 6/9 patients [66%]), confusion (2 ×), meningism (1 ×), vomiting, headache, somnolence (1 ×), stupor and coma (1 ×), and spastic hemiplegia (1 ×) were noted. Brain MRI sowed periventricular lesions (1 ×), lesions in the centrum semiovale (1 × , bilateral), lesions in the cerebral white matter (WM) (1 ×), a lesion in the occipital WM (1 ×), lesions in the basal ganglia (2 × ; bilateral in both), lesions in the thalamus (4 × ; with a pulvinar sign in one; bilateral in at least three), a tumefactive frontal lesion (1 ×), subcortical lesions (1 ×), and lesions in the frontal cortex (3 ×); in three further cases lesions in “supratentorial regions” (1 ×), “bilateral lesions in the fronto-parietal region”, or just “multiple brain lesions” (1 ×) were reported. Some of the lesions were described as being “poorly demarcated”, “fluffy” or “ill-defined”. Contrast enhancement was reported just in two cases and was absent in both.

#### Attack severity

Severe attack-related manifestations (functional blindness, tetraparesis, paraplegia, hemiplegia, disturbed consciousness) were present in at least 13/20 (65%), in 16/20 (80%) if urinary retention and other forms of bladder/bowel dysfunction are also considered to qualify as severe manifestations, and in 17/20 (85%) if also BST involvement is considered to qualify as severe. In the remaining patients, reduced visual acuity (0.5 or 0.63, respectively) and fever and vomiting followed by a sensory mid-thoracic level were present.

#### Treatments

The most commonly used therapy for acute attacks was high-dose intravenous methylprednisolone (IVMP) (mostly 1 g/day over 5 days, less often over 3 days), but escalatory treatment with plasma exchange (PLEX) was deemed necessary in nine patients. In some patients, several courses of IVMP were given or IVMP treatment was prolonged (1 × cumulative dose 11 g, followed by PLEX [7]; 1 × 7 g followed by PLEX [36]; 1 × 6 g [30]). Oral steroids (OS) were used at varying dosage in 15/20 (75%) patients (after IVMP in all cases), with dosages usually reduced gradually (depending on tapering strategies, over a period of up to 12 months). Tapering was associated with flaring up of symptoms in at least one case [7]. Tocilizumab (TCZ), an IL-6 receptor blocker, was used as escalatory and as long-term treatment over a period of 3 months in one patient [7], and long-term immunosuppressive treatment with mycophenolate mofetil (MMF) was initiated in another patient.

#### Treatment outcomes

Complete recovery was achieved after IVMP treatment in a patient with isolated unilateral MOG-ON (present case), and good recovery in a patient with isolated BSTE [30], both of which had occurred after vaccination with an mRNA vaccine. Almost complete recovery was also reported in two patients with myelitis or myelitis plus STE after ChAdOx1 nCoV-19 [9].

In 11 further ChAdOx1 nCoV-19-vaccinated patients with different manifestations, improvement was considered “significant” (in 10) [36] or “good” (in one) [32]. All of these were treated with IVMP, nine in addition with oral corticosteroids, six in addition with PLEX, and one was treated with MMF in addition to IVMP and oral steroids. However, severity and type of residual symptoms in these patients were not reported.

In one patient with myelitis, spastic tetraparesis resolved following treatment with PLEX (five cycles), IVMP (1 g/d for 5 days) and oral corticosteroids, but urinary incontinence were noted 135 days after onset [9]; in a patient with myelitis and encephalitis, paraplegia, urinary retention and encephalopathic symptoms resolved after treatment with IVMP (1 g/d for 3 days), intravenous immunoglobulin (IVIG) (2 g/kg for 5 days) and oral corticosteroids but sensory impairment of the lower limbs remained present 118 days after onset [9]; another patient recovered from LETM and encephalitis with tetraparesis and coma only after several courses of IVMP (11 g in total) and escalatory therapy with PLEX (seven sessions) but flaring-up of symptoms occurred during tapering of corticosteroids (as often seen in MOG-EM [20]) and persistent mild cerebellar symptoms with predominant intention tremor of the right arm were noted 3 months after onset despite continuous treatment with TCZ and oral prednisone [7]; one patient recovered from myelitis-associated sensory symptoms after IVMP and PLEX but had a residual gait disturbance and still needed urinary catheterisation at last follow-up [6]; and finally, one patient recovered from LETM with paraparesis after IVMP treatment (1 g/d for 5 days) but urinary retention persisted 3 months after onset [34]. All of these patients had been vaccinated with ChAdOx1 nCoV-19.

Overall, 9/20 patients (45%) needed escalatory treatment with PLEX, several cycles of high-dose IVMP therapy had to be given because of persisting symptoms or flaring-up of symptoms in at least three cases, and additional immunosuppression with TCZ or MMF was carried out in three. Complete or almost complete recovery was explicitly reported only for two patients, but substantial improvement was obviously achieved in the majority of cases at last follow-up (median 44 days after onset, range 5–180; > 30 d in 11 cases). These data suggest a relatively severe disease onset in postvaccinal MOG-EM. However, a potential reporting bias towards more complicated cases cannot be ruled out.

MRI outcomes were reported for only few patients. Repeat MRI at 3 months showed resolution of an occipital WM lesion after treatment with high-dose IVMP, PLEX and OS, but persistent SC lesions, in one patient [9]; complete resolution of the brain and almost complete resolution of the SC lesions after high-dose IVMP in another patient [7]; and only partial resolution of SC, subcortical WM and pulvinar lesions after several courses of high-dose IVMP, PLEX, OS and TCZ in a third patient [7]. In a fourth patient, MRI was repeated after two courses of IVMP and showed a shrinking CP lesion with decreased contrast enhancement after high-dose IVMP [30].

#### Serology

**Fig. 1:**
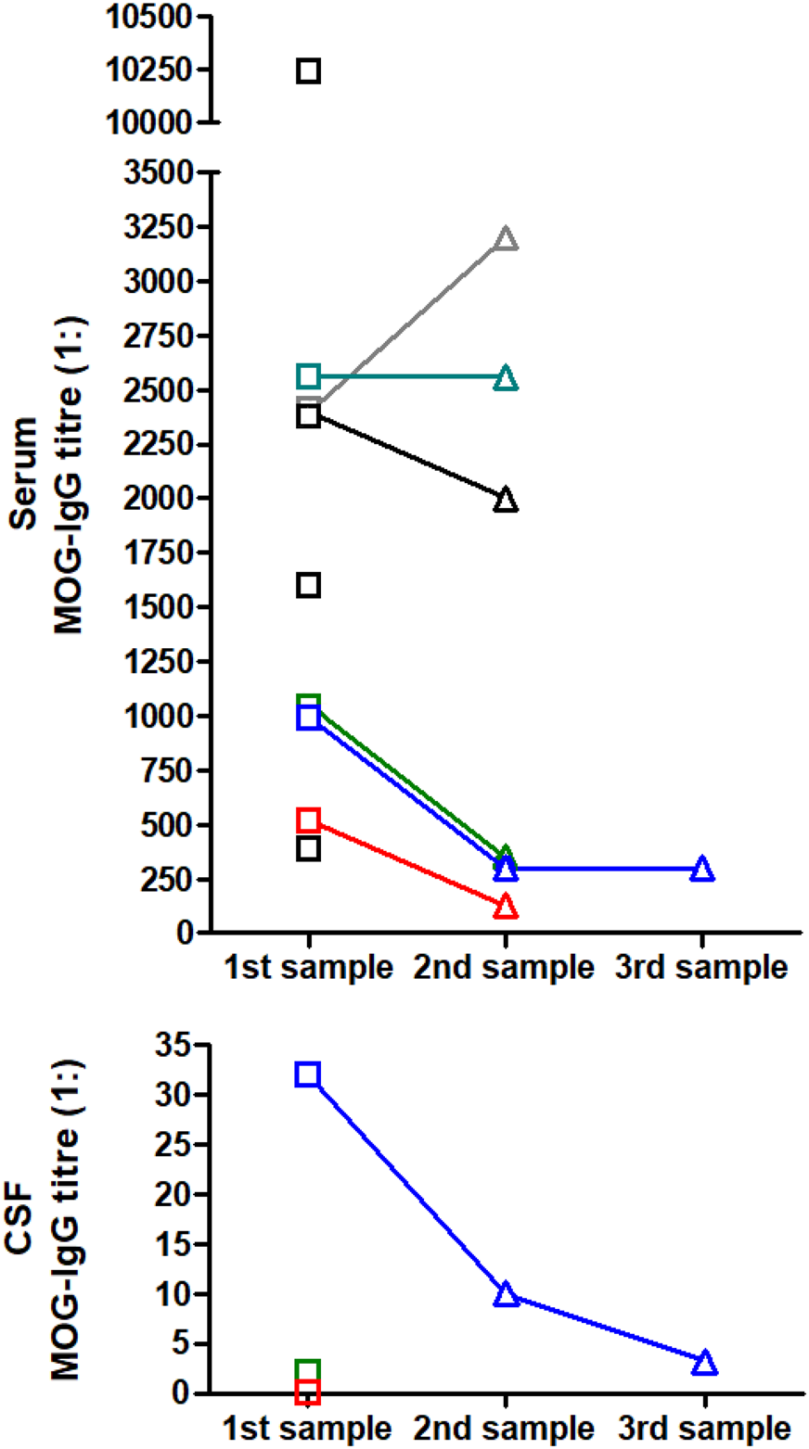
Serum and CSF MOG-IgG titres in nine patients with newly emerging MOG-EM after vaccination with SARS-CoV-2 (titres not reported for the remaining 11 patients). Follow-up results were available from 7 patients; in all of these, MOG-IgG remained detectable at second or third determination (follow-up 81 [present report, green], 90 days [case 18, blue], 90 days [case 20, dark cyan], and 180 days [case 13, red], respectively, in four patients, and “2 to 12 weeks” after first testing in the remainder), at high titres in some patients. Note that in one case, the serum MOG-IgG follow-up result was reported as “positive” but no titre given (case 16). CSF MOG-IgG titres were reported in three cases (lower panel; matched CSF and serum samples are shown in matching colours); in one further patient, CSF MOG-IgG (as serum MOG-IgG) was classified as “strongly positive” but titres were not stated (case 3)

Cell-based assays (CBA) employing full-length human MOG protein were used for MOG-IgG testing in at least 18/20 patients (no data in 2, although the cut-off given suggests use of a live CBA in one of them) in accordance with current diagnostic recommendations [14, 15]. Peak serum MOG-IgG titres at first testing ranged between 1:400 and 1:10,240 (median, 1:1600; *N* = 9 with available data) and were all clearly positive according to assay-specific cut-offs (the individual results for each patient and assay-specific cut-off values are given in the Table 1). Titres were not specified in 11 cases but described as “strongly positive” in 6 of them. In all patients with available follow-up data (*N* = 6), MOG remained detectable over time (median 1:1160, range 256–3200) despite treatment with high-dose IVMP (1 × stable titre of 1:2560 after 3 months [34], 1 × 1:512 → 1:256 after 3 months [30]); or IVMP and PLEX [9]; or several courses of IVMP, PLEX, OS and TCZ (1 × minor decline from initially 1:1000 to 1:320 3 months later) [7]; with IVMP and OS (1 × 1:1000 → 1:320 almost 3 months later [present case]; 1 × 1:2400 → 1:2000[9]; 1 × 1:1600 → positive at unspecified titre); or with IVMP, OS and IVIG (1 × 1:2500 → 1:3200) [9] (Fig. 1). In one of these (treated with high-dose IVMP), MOG-IgG was still detectable in a Fc-specific CBA 6 months after onset but not in an IgG1-specific CBA.

#### CSF findings

CSF pleocytosis defined as > 5 cells/µl was present in 11/14 patients (79%; no data in 6) and was predominantly mononuclear (lymphocytic/lymphomonocytic) in most (Table 1). Neutrophils were present in at least two patients, which was the predominant cell type in one of them. The median peak CSF WCC was 44 cells/μl (range 0–720; interquartile range 7–109; *N* = 14; > 100 cells/μl in 5/14 [35%]; median peak WCC 58 cells/μl among those with pleocytosis). The CSF WCC was low in the only patients with isolated ON (6 and 0 cells/µl, respectively) or isolated BSTE (< 5 cells/µl), which compared with a median of 82 cells/µl among patients with MY (± involvement of other CNS areas; *N* = 10 with available data). The highest cell counts were observed in a patient with myelitis and BST involvement (720 cells/µl; predominantly lymphocytic) and in a patient with myelitis, BST involvement and STE (545 cells/µl at onset → 720 cells/µl 5 days later; predominantly granulocytic). The median peak CSF WCC was higher than that observed in patients with acute disease in a large cohort study on CSF findings in MOG-EM [19] (44 cells/μl vs. 9 cells/μl). This may, at least in part, reflect the substantially higher proportion of patients with myelitis (which is generally associated with higher CSF WCCs in MOG-EM than other manifestations [19]) in the present series. However, the difference between the two cohorts did not reach statistical significance, irrespective of whether all patients (*p* = 0.21; Mann–Whitney U test) or only patients with acute myelitis at the time of lumbar puncture (median 82 cells/μl vs. 39 cells/μl in [19]; *p* = 0.369) were included in the analysis. CSF-restricted OCB were observed in 3/8 patients (1 × bilateral ON plus but limb spasticity, 1 × bilateral ON plus myelitis and infra- and supratentorial brain involvement, 1 × isolated BSTE; MOG-IgG serum titres were clearly positive in all of these) but were not reported in the majority of patients; at least one additional patient had identical OCB in the CSF and serum, indicating systemic inflammation. Peak CSF total protein (TP) levels were elevated in 7/14 (50%) patients (based on an upper reference limit of 0.45 mg/l [40]; no data in 6). The median CSF TP concentration was 47 mg/dl (range 18–144) among all patients with available data (*N* = 13) and 64 mg/dl (range 47–144) among those with elevated levels. CSF L-lactate levels were reported only in three patients and were normal in one, high in another (2.5 mmol/l at first and 2.0 at second lumbar puncture; age specific cut-off: 2.6) and clearly increased in the third patient in two samples (4.4 and 3.6 mmol/l 5 days later; age specific cut-off: 2.1) but normal in a third sample (1.8 mmol/l after 3 months). Interestingly, granulocytes, which are a known source of CSF L-lactate, were substantially elevated in the two samples with elevated CSF L-lactate levels.

#### Differential diagnosis

Alternative diagnoses were explicitly excluded in many but not all cases and the extent of differential diagnostic investigations varied among reports (Table 1). However, as mentioned above, clinical and radiological features were compatible with MOG-EM in all cases and MOG-IgG titres were sufficiently high in most cases with available data not to cast substantial doubts on the diagnosis, remained detectable over time in those tested, and were determined by means of CBA in most patients (Table 1). If, taking a very strict stance, one excludes those cases in whom titres were not reported (1 × after BNT162b2) or titres were not classified as “strongly elevated” (4 × after ChAdOx1 nCoV-19 [36]), still 15 unequivocal cases of MOG-EM would remain. AQP4-IgG-positive NMOSD was excluded in 8/20 patients; the AQP4-IgG serostatus was not mentioned in the remainder.

Notably, MOG-EM mimicked bacterial CNS infection or early-stage viral infection in one patient with predominantly granulocytic CSF pleocytosis (545–720 cells/µl during acute disease) and elevated CSF L-lactate levels [7]. Extensive evaluation for infection, including next generation sequencing (NGS) for over 1500 pathogens, was negative. This is in line with previous reports on a relatively high proportion of granulocytic CSF pleocytosis (around 45% in adults; more common in patients with SC or brain involvement than in patients with isolated ON) and CSF L-lactate elevation in MOG-EM both in adult and paediatric patients by ourselves and others [13, 19, 20] (which is similar to AQP4-IgG-positive NMOSD [16, 22]). While CSF L-lactate concentrations exceeded the age-dependent reference range in 26% of all cases in a large recent study on adult patients with MOG-EM, CSF L-lactate concentrations > 3 or > 4 mmol/l, as seen in a majority of patients with acute bacterial meningitis, were absent in 90% and 98% of cases, respectively. The high CSF L-lactate levels observed in this patient are thus unusual but per se do not exclude the diagnosis. MOG-IgG titres were highly positive (1:1000; cut-off ≥ 1:10) at onset and remained detectable (1:320) in two follow-up samples taken over a period of 3 months [7].

#### Personal and family history

In none of the existing reports was a personal or family history positive for autoimmune or neurological disorders mentioned except for an episode of vertigo 4 months earlier in one patient which is at least hypothetically compatible with pre-existing MOG-EM (although cranial MRI was negative at the time vertigo occurred). One patient had a history of allergic asthma.

#### Re-exposure

In one patient with a severe MOG-EM after vaccination with ChAdOx1 nCoV-19, a second vaccination was given after clinical recovery and under OS therapy (100 mg/d for 5 days), this time using a (non-specified) mRNA vaccine. This was followed by a significant increase in antibody titres but no relapse.

## Discussion

Overall, we identified 20 patients with clinical onset of MOG-EM after vaccination against SARS-CoV-2. Several observations are of interest from a clinical point of view: First, postvaccinal MOG-EM possibly takes a more severe disease course compared with non-vaccination-related MOG-EM, at least at onset of disease, as indicated by the relatively widespread CNS involvement mentioned above; the high rate of SC (75%) and BST (50%) lesions; the high SC lesion load in those with signs of myelitis (with LETM in at least 9/15; multiple SC lesions, including multiple LETM lesions, in at least 6/15 patients; and exceptionally long LETM lesions in at least 5/15 patients, reaching from the cervical or dorsal SC to the conus in at least 4 cases); the high rate of patients experiencing functional blindness (50% of those with ON), tetraparesis, paraplegia or bladder/bowel dysfunction (13/20 patients); and the fact that escalatory PLEX was required in 45% of all cases and multiple courses of IVMP in some. However, as mentioned above, a reporting bias towards more severe cases cannot be fully excluded, patient numbers are still too low to draw definite conclusions, and in around 30% of cases only one CNS region was affected. Prospective data and rigorous comparison with non-vaccination-associated cases from the same population are needed to confirm the observed association of vaccination-associated cases with more widespread CNS involvement. 

Second, all patients reported so far were older than 25 years at onset (range 28–68). However, the fact that approval for use of SARS-CoV-2 in children was obtained only relatively recently, may partly explain why no paediatric cases have yet been documented. It is thus possible that paediatric cases will be reported in the future. Given the high proportion of MOG-IgG-associated cases among cases of CNS demyelination in children, it is highly advisable to test children presenting with symptoms compatible with MOG-EM for that antibody. Whether immunological differences between adults and children may also be involved is unknown.

Taking into account that six patients were > 50 years of age (two of them > 65) at onset in the present series, MOG-EM should be considered also in older patients presenting with newly emerging neurological symptoms after vaccination against SARS-CoV-2. In fact, the median age at onset in the present series (43.5 years) was higher than that observed in previous adult MOG-EM cohorts that included no SARS-CoV-2 vaccination-associated cases [5, 20, 43], possibly reflecting the fact that the vaccination campaign initially focused on the elderly and that in many countries the absolute number of adults ≥ 40 years of age vaccinated to date clearly exceeds that of vaccinated adults < 40 years of age (e.g. [8, 31, 35.]). Postvaccinal MOG-EM may occur both in women and in men, with no marked female or male preponderance.

Third, while some attacks were difficult to control by high-dose IVMP alone or symptoms flared up during oral tapering in some patients and although escalatory treatment with PLEX was required in many cases (in one patient each, TCZ and IVIG were given in addition), at least partial recovery was achieved in most patients and complete recovery in some. Early escalatory treatment with PLEX is generally recommended in acute attacks of MOG-EM that do not respond immediately and seems to be effective also in SARS-CoV2-vaccination-associated cases based on the relatively few reports available to date.

Fourth, repeat MRI during the acute phase may be advisable, since the initial MRI may markedly underestimate the peak lesion load if obtained early during the disease course as suggested by a substantial increase in lesions in both of the only two cases with early follow-up MRIs (5 days after onset).

Fifth, it should be kept in mind that relapses in MOG-EM may still occur many months or years after onset [20]. Of note, MOG-IgG was still detectable at follow-up examinations up to 6 months after onset in 7/7 patients tested, suggesting a persisting risk of relapse. If seroconversion, i.e., disappearance of the antibody, is taken as a reason not to start (or, later in the disease course, to end) immunosuppressive treatment, it needs to be considered that seroconversion can be transient, i.e., antibody titres may temporarily fall below the cut-off but rise again later, and MOG-IgG should be monitored closely in such patients so not to overlook reappearance of MOG-IgG, which entails the risk of future relapse. More has to be learned about the rate of seroconversion in SARS-CoV-2 vaccination-associated MOG-EM and about the long-term risk of relapse in this special setting.

This said, it is important to understand that assay sensitivity varies and false-negative results may occur. In case 13, MOG-IgG was still detectable in an Fc-specific assay 6 months after onset but was (falsely) negative in an IgG1-specific assay. Although assays using Fc-specific secondary antibodies to detect patient IgG are not per se preferential, since assay sensitivity depends on many more factors, this case highlights the potential risk of underdiagnosing MOG-EM and of overestimating the rate of seroconversion in MOG-EM when using IgG1-specific assays (a type of assay that detects solely MOG-IgG of this specific subclass, which, however, accounts only for a varying proportion of MOG-IgG present in a patient’s serum) instead of Fc-specific assays (which, ideally, detect MOG-IgG of all IgG subclasses).

Sixth, routine CSF findings did not differ significantly from those in patients with non-vaccination-associated MOG-EM in almost all cases [13, 19, 42]. In case 18, neutrophilic CSF pleocytosis with a relatively high CSF WCC was noted. Neutrophils have been previously observed in 40–70% of patients with MOG-IgG, both in adults [19] and in children [13], and do thus not argue against a diagnosis of MOG-EM. However, concomitant infection should nonetheless be ruled out, especially (but not only) if the WCC is unusually high. In patient 18, peak WCC (720 cells/µl) exceeded the maximum WCCs (463 cells/µl and 256 cells/µl) seen in two large previous studies on adult patients and paediatric patients, respectively, with MOG-EM [13, 19], but extensive screening for bacterial and viral diseases was negative. For this study, we assessed for the first time the MOG-AI in postvaccinal MOG-EM. However, as in non-vaccination-associated cases, no indication for intrathecal synthesis was found in our patient.

We also performed for the first time IgG subclass analyses in SARS-CoV-2-vaccination-related MOG-EM. The fact that exclusively MOG-IgG of the strongly complement-activating IgG1 subclass was detected in the CSF and predominantly MOG-IgG1 also in the serum supports a potential pathogenic role of MOG-IgG in our patient.

Seventh, in none of the patients was a positive personal or family history for allergies, autoimmune diseases or neurological disorders reported (with the exception of single episode of vertigo in one patient). As a limitation, underreporting cannot be excluded given that no explicit information was provided for some patients.

While all patients formally met the recently proposed criteria for a “probable” causal relationship between SARS-CoV-2 vaccination and neurological complications [3] and the criteria for “possible” or “likely” causality according to the WHO-UMC system for standardised case causality assessment [46], it is important to stress that formal immunological evidence proving a pathophysiological link (e.g., by demonstrating epitopes involved in molecular mimicry) is missing so far.

Provided the two events are pathophysiologically linked, vaccination could either have newly induced the disease (e.g., due to molecular mimicry/epitope spreading or inflammation-related demasking/release of MOG epitopes) or, alternatively, acted as an unspecific trigger causing clinical exacerbation of pre-existing subclinical MOG-EM. The latter notion is at least conceivable when one considers that other antibodies, such as AQP4-IgG in NMOSD [17, 27, 33, 37], have been retrospectively found in stored serum samples obtained months to many years prior to clinical disease onset. Finally, a temporal coincidence of the two events also cannot be excluded given the currently high vaccination numbers worldwide. Rigorous population-based data on the incidence of postvaccinal and non-vaccination-associated MOG-EM would be important to answer this question but are currently not available.

However, two findings seem to argue against a simple temporal coincidence:

First, it is notable that most documented cases occurred after previous vaccination with the vector-based Oxford/AstraZeneca vaccine ChAdOx1-S/ChAdOx1 nCoV-19 (18/20 patients, 90%), despite the fact that vastly more patients have been immunized with other vaccines worldwide, suggesting some sort of a causal relationship with ChAdOx1-S/ChAdOx1 nCoV-19. This association is strengthened by the fact that no cases have been reported from the United States and China, where the ChAdOx1-S/ChAdOx1 nCoV-19 vaccine has not yet been approved, despite extensive research on both MOG-EM and COVID-19 being performed in these two large countries with relatively high vaccination rates. Onset of MOG-EM after vaccination with the Pfizer-BioNTech mRNA vaccine (BNT162b2) has been described only in two patients worldwide, one of them the patient presented here in detail (who had previously received two doses of ChAdOx-1-S), and only one case with onset after vaccination with the Moderna mRNA vaccine 1273 has been reported. Finally, re-vaccination with a (non-specified) mRNA vaccine in a patient with severe MOG-EM after initial vaccination with ChAdOx1 nCoV-19 did not result in relapse in the only patient reported so far[7].

Of note, vaccination with ChAdOx1-S/ChAdOx1 nCoV-19 is associated with an increased risk for other antibody-related diseases, such as Guillain–Barre syndrome [39] and platelet factor 4 (PF4) antibody-related vaccine-induced thrombosis and thrombocytopenia (VITT) [10].

The preponderance of ChAdOx1-S/ChAdOx1 nCoV-19-associated cases should result in consideration of a role also for the vector (rather than the SARS-CoV-2 spike protein used as immunogen) and of adjuvants in future studies investigating the aetiopathogenesis of MOG-EM after SARS-CoV-2 vaccination. Besides molecular mimicry, also indirect effects need to be considered. In VITT, binding of the adenovirus vector to cellular heparan sulphate proteoglycan (HSPG) (subsequently resulting in thrombocyte-activating PF4 immune complexes with heparan and/or heparin) has been recently proposed to underly the pathogenesis of this complication of vaccination with ChAdOx1-S/ChAdOx1 nCoV-19. It is also interesting in this context that BNT162b2 was given, as a booster shot, after two previous vaccinations with ChAdOx1-S in our patient. Considering the observed strong association of MOG-EM with ChAdOx1-S/ChAdOx1 nCoV-19, it is at least conceivable (although necessarily speculative) that MOG antibody synthesis was prompted already by the first two vaccinations with ChAdOx1-S, while the BNT162b2 booster only triggered disease exacerbation (e.g., by causing inflammation-derived blood brain-barrier disruption [13, 19], T cell activation, and a systemic and intrathecal proinflammatory environment).

As a limitation, however, no reports on MOG-EM after SARS-CoV-2 vaccination exist also from several countries in which ChAdOx1-S/ChAdOx1 nCoV-19 has in fact been approved. A reporting bias can thus not be fully ruled out. On the other hand, MOG-IgG testing is not available in some regions of the world. Prospective data that take into account both vaccination rates with the various vaccines and incidence of vaccination-associated cases of MOG-EM within one and the same population are needed to definitely confirm the observed preponderance of ChAdOx1-S/ChAdOx1 nCoV-19.

Second, a high proportion of patients exhibited clinical or radiological involvement of more than one CNS area (SC, BST, STB, optic nerve). This is in marked contrast to classical MOG-EM, in which isolated ON is considered the most frequently occurring presenting manifestation in adults. However, ADEM-like presentation is a common and well-known feature in patients with postvaccinal CNS demyelination in general. In fact, more than one CNS region was also affected in many patients previously reported by ourselves and others with postvaccinal onset of MOG-EM (1 × 2 weeks after revaccination against diphtheria, tetanus, pertussis, polio, and influenza [20], 1 × 2 weeks after vaccination against diphtheria, tetanus, and pertussis [20], 1 × 3 and 2 weeks, respectively, after first vaccination against tetanus and varicella zoster virus and 2 weeks after re-vaccination against measles, mumps, and rubella [25], 1 × 2 weeks after vaccination against pertussis [41], 1 × after second vaccination against measles and rubella and booster vaccination against Japanese encephalitis [1]), although not in all (1 × isolated LETM 2 weeks after vaccination against diphtheria, pertussis, poliomyelitis and tetanus [28], 2 × ON 11 days and 3 weeks, respectively, after vaccination against *Herpes simplex* [47] and after an unspecified vaccination [28], respectively; as a limitation, no data on radiological involvement of the brain and/or spinal cord was provided in these cases, leaving the possibility that more than one area was affected subclinically also in these patients, and MOG-IgG titres were very low in one of them [47]). The presence of more widespread, ADEM-like CNS involvement at onset in the majority of patients in the present series (70%) thus at least supports the notion of MOG-EM having occurred as a complication of SARS-CoV-2 vaccination, at least in those presenting with such phenotype. As a limitation, it does per se not prove such a relationship, all the more as ADEM occurs also, and much more often, in postinfectious settings, including after minor respiratory infections, the presence or absence of which in the weeks before onset was not stated in most reports.

The fact that vaccinations against different viruses have been reported in association with newly emerging MOG-EM, on the other hand, would argue in favour of (but not prove) the notion that vaccination is a non-specific trigger rather than the specific cause of MOG-directed autoimmunity. This notion is further supported by the fact that vaccination with the Oxford AstraZeneca vaccine and, more rarely, other vaccines (BBV152, “inactivated vaccine”) has also been reported before a first attack of AQP4-IgG-positive NMOSD [2, 4, 36] and other forms of CNS demyelination not associated with MOG-IgG [24, 36, 38, 44, 48]. ChAdOx1-S/ChAdOx1 nCoV-19 might thus just be a more potent trigger than BNT162b. However, it is still possible that the vaccination is the actual cause (i.e., starts an autoimmune reaction) in one disease and just a (non-specific) trigger in other conditions. Moreover, the actual mechanism may differ between vaccines and even interindividually (e.g., depending on HLA types and other genetic differences between patients).

We count the number of items analysed, which included clinical, radiological, serological and other laboratory features, vaccination type and timing, and treatment outcomes, among the strengths of this study. Potential limitations include the retrospective design, a possible reporting bias in favour of more severe cases, and, most importantly, the fact that no control group was available because the cases analysed were reported by various centres worldwide. As discussed above, symptoms started already after the first vaccination in the majority of cases but only after the second or third vaccination in four patients. This gives rise not only to the question of whether pre-existing subclinical MOG-EM may have existed in some cases and been triggered by vaccination-induced inflammation but also whether previous contact with the SARS-CoV-2 spike protein plays any role in disease pathogenesis. Although no or no remarkable medical history was explicitly reported for most patients, it is a limitation that previous or concomitant COVID-19 was either not properly excluded (e.g., by testing for SARS-CoV-2 nucleocapsid protein-specific antibodies) or, provided it was excluded, the relevant data were not included in most case reports and series analysed. Given the high prevalence of COVID-19, the high proportion of asymptomatic cases, and the deficient political measures to prevent spread of infection in many countries (including the countries of origin of all patients included in the present analysis), it is at least conceivable that prior or, less likely (since hospitalised patients usually get tested for acute SARS-CoV-2 infection), concomitant infection unrecognized by the patients may have occurred in individual cases. In that case, the vaccination would not have been the first encounter with the antigen, which may be of relevance when it comes to understanding the pathogenesis of postvaccinal MOG-EM in general as well as the apparently elevated severity compared with non-vaccine-associated MOG-EM at onset. Previous infection with SARS-CoV-2 was explicitly reported in only one patient, who had suffered from COVID-19 nine months prior to vaccination (case 5 in the Table 1) and developed a severe attack of MOG-EM with myelitis (with swelling of the SC and paraplegia), BST and STE (diagnosed as “ADEM”) 10 days after the first dose of ChAdOx1 nCoV-19 requiring IVMP, PLEX, OS and immunosuppressive treatment with MMF.

Complications of vaccination are being systematically documented in official registries in many countries. However, we believe it is crucial also to include all of these patients in academic registries and biorepositories for use in studies striving to improve our understanding of the observed association of autoimmune neurological disorders, such as MOG-EM, with vaccination against or infection with SARS-CoV-2. In Germany, the Neuromyelitis optica Study Group (NEMOS) currently collects data on cases of MOG-EM and NMOSD associated with either SARS-CoV-2 vaccination or COVID-19 (www.nemos-net.de), and the German Network for Research on Autoimmune Encephalitis (GENERATE) documents cases of other forms of encephalitis/encephalomyelitis of putative autoimmune aetiology occurring in these settings (www.generate-net.de). Similar initiatives exist in other countries. Readers are strongly encouraged to include eligible patients in these registries.

We consider it important to stress that, on the basis of the existing literature and the fact that more than 11 billion vaccine doses have been given worldwide, vaccination against SARS-CoV-2 seems to be associated with newly emerging MOG-EM only extremely rarely, and that a causal link between the two events has not yet been formally demonstrated. The benefits of vaccination, which is highly effective in preventing severe COVID-19, clearly outweigh the minimal risk of developing MOG-EM.

Given the therapeutic and prognostic implications of MOG-IgG seropositivity, we strongly recommend that all patients presenting with clinical symptoms compatible with acute optic neuritis, encephalitis, brainstem encephalitis, or myelitis (or any combination thereof) after vaccination against SARS-CoV-2 and radiological or, only in the case of ON, electrophysiological signs of CNS demyelination should be tested for serum MOG-IgG without delay by means of a well-established CBA employing full-length human MOG as test substrate and Fc- (or IgG1-)specific anti-human IgG secondary antibodies [14]. If available, all positive test results should be confirmed using a second assay, especially in the event that so-called ‘red flags’ as listed in [14] are present. As sensitivity differs between assays, re-testing should be considered in case of a negative (especially if borderline negative) test result if MOG-EM is still suspected. Acute treatment should follow published recommendations and guidelines. If high-dose IVMP does not lead to rapid and complete or almost complete resolution of symptoms, escalatory PLEX is often required; sometimes IVIG is used, especially in children [11, 26]. It should be noted that MOG-EM-associated symptoms may be steroid-dependent; OS therapy with tapering is therefore commonly used after IVMP. Whether long-term immunosuppressive/immunomodulatory therapy (often with azathioprine, rituximab, MMF or, especially in children, IVIG) is required depends on clinical presentation, attack severity, the degree of symptom resolution after acute therapy, and on whether relapses occur [11, 26, 45]. More needs to be learned about the long-term treatment needs of patients with postvaccinal MOG-EM.
